# The quest for optimal ketamine dosing formula in treatment-resistant major depressive disorder

**DOI:** 10.1007/s43440-024-00637-x

**Published:** 2024-08-26

**Authors:** Julia Kwaśna, Wiesław Jerzy Cubała, Aleksander Kwaśny, Alina Wilkowska

**Affiliations:** Department of Psychiatry, Faculty of Medicine, Medical University of Gdańsk, Gdańsk, 80-214 Poland

**Keywords:** Major depressive disorder, Ketamine, Dose calculation, Lean body mass, Ideal body weight, Body surface area

## Abstract

**Background:**

Emerging evidence indicates that intravenous ketamine is effective in managing treatment-resistant unipolar and bipolar depression. Clinical studies highlight its favorable efficacy, safety, and tolerability profile within a dosage range of 0.5-1.0 mg/kg based on actual body weight. However, data on alternative dosage calculation methods, particularly in relation to body mass index (BMI) and therapeutic outcomes, remain limited.

**Methods:**

This retrospective analysis of an open-label study aims to evaluate dose calculation strategies and their impact on treatment response among inpatients with treatment-resistant major depressive disorder (MDD) (*n* = 28). The study employed the Boer and Devine formulas to determine lean body mass (LBM) and ideal body weight (IBW), and the Mosteller formula to estimate body surface area (BSA). The calculated doses were then compared with the actual doses administered or converted to a dosage per square meter for both responders and non-responders.

**Results:**

Regardless of treatment response, defined as a reduction of 50% in the Montgomery-Åsberg Depression Rating Scale, the use of alternative ketamine dosing formulas resulted in underdosing compared to the standardized dose of 0.5 mg/kg. Only two participants received higher doses (102.7% and 113.0%) when the Devine formula was applied.

**Conclusions:**

This study suggests that ketamine dosing formulas, alternative to the standardized 0.5 mg/kg based on body weight, may lead to underdosing and potentially impact outcome interpretation. To enhance dosing accuracy, future studies should consider incorporating body impedance analysis and waist-to-hip ratio measurements, as this study did not account for body composition.

## Introduction

Major depressive disorder (MDD) is estimated to affect over one-fourth of the general population over their lifetime [1]. Increasing evidence supports the effectiveness of intravenous (IV) ketamine – an N-methyl-d-aspartate (NMDA) receptor antagonist — as an intervention for treatment-resistant unipolar (TRD) and bipolar depression (TRD-BP) [2, 3].

Subtherapeutic amounts of ketamine have displayed the potential for swiftly addressing TRD patients [4–7]. The consensus guideline concerning ketamine hydrochloride usage proposes that dosing should correlate with overall body weight [8]. Nonetheless, observations highlighting notable cardiovascular alterations in overweight individuals advocate for tailoring ketamine dosage among those with elevated body mass index (BMI) to align with their ideal body weight [8]. This theory speculates that the immediate reaction may stem from heightened ketamine doses administered to individuals with higher BMI, while the absence of an enduring response could be linked to metabolic syndrome [9]. Furthermore, the effectiveness of ketamine appears to be influenced by the cumulative dosage administered, showing enhanced responses in patients with greater adiposity [10].

Most studies follow the ketamine dosing regimen of 0.5 mg/kg in 40 min long infusion [11]. However, some researchers advocate for dose titration within the range of 0.5–0.75 mg/kg, suggesting a higher likelihood of therapeutic response without a significant increase in adverse effects [12]. Fava et al. (2020) aimed to ascertain the optimal ketamine dosage and found evidence supporting the efficacy of subanesthetic IV doses of ketamine at 0.5 mg/kg and 1.0 mg/kg. However, they did not observe clear or consistent evidence of clinically significant efficacy at lower doses (0.1 and 0.2 mg/kg). This indicates an unmet need for an optimal ketamine dosing regimen [13].

This retrospective study explores whether dosing calculations based on actual body weight, lean body mass (LBM), ideal body weight (IBW), or body surface area (BSA) are associated with the response to ketamine treatment in patients with treatment-resistant MDD. There is an unmet need for consensual IV ketamine administration being supported by the robust clinical data.

## Methods

### Patients

This is a post-hoc analysis of a study cohort involving individuals from an IV ketamine naturalistic observational registry protocol, focusing on the safety and tolerability of ketamine in TRD. Hospitalized patients within a tertiary medical center (Medical University of Gdańsk) diagnosed with a depressive episode within the course of MDD were included. Participants were classified by a clinician psychiatrist by the Diagnostic and Statistical Manual of Mental Disorders, Fifth Edition (DSM-5) criteria. All participants demonstrated resistance to treatment in the current depressive episode, defined as an inadequate response to at least two antidepressants at appropriate doses and duration. Only medically stable adults were enrolled in the study. Patients were allowed to continue their current medications during ketamine treatment if necessary. Exclusion criteria encompassed pregnancy, breastfeeding, an active history of uncontrolled diseases, or previous adverse reactions to ketamine. The study population and methodology are detailed more precisely elsewhere (Słupski et al. [14]). This study was registered at ClinicalTrials.gov (NCT04226963) and received approval from the Independent Bioethics Committee for Scientific Research at the Medical University of Gdańsk, Poland (NKBBN/172–674/2019). Written informed consent for participation and data use was obtained from all patients.

### Study design

The study employed an observational design where patients continued their baseline psychotropic treatment and necessary management for chronic somatic conditions during the ketamine infusions. The therapeutic intervention included eight IV ketamine infusions administered over four weeks as an adjunct to standard care. Eight ketamine infusions were given at a dose of 0.5 mg/kg, adjusted to the patient’s actual body weight, via IV infusion over 40 min twice per week. Safety oversight was provided by the managing psychiatrist, who monitored patients’ safety indicators, including vital signs, before, during, and after the infusion.

Throughout the study, the principle of single patient and single rater was maintained, ensuring that each clinician-administered psychometric scale was applied consistently by the same rater for each individual. Patients were assessed by the clinician using the Montgomery-Åsberg Depression Rating Scale (MADRS) at screening, before 3rd, 5th, 7th ketamine infusion and 7 days post-treatment. The primary endpoint was the change in the MADRS score after the seventh ketamine infusion. A response was defined as a 50% or greater reduction in the MADRS score from baseline.

### Dose calculations

Three distinct formulas for dosage calculation, based on various factors, were employed to determine appropriate dosing regimens. The Mosteller formula, a widely used method, estimates body surface area (BSA) using the equation: BSA = √[(height (cm) * weight (kg)) / 3600]. The administered dose was then divided by the patient’s BSA to calculate the dosage per square meter.

Another formula calculates lean body mass (LBM), representing body weight minus fat weight, such as the Boer formula. LBM dosage calculation differs between males and females. For males, the estimated LBM is 0.407(weight) + 0.267(height) – 19.2, while for females, it is 0.252(weight) + 0.473(height) – 48.3.

The third calculation method is based on ideal body weight (IBW), an estimate of healthy weight based on height. An example of IBW estimation is the Devine formula. For males, the equation is IBW = 50 kg + 0.9 kg × (height (cm) − 152); for females, it is IBW = 45.5 kg + 0.9 kg × (height (cm) − 152). Theoretical doses for the Boer and Devine formulas were calculated based on a regimen of 0.5 mg/kg.

### Statistical methods

In our study, we did not perform any statistical analyses; rather, we conducted simple computations using the aforementioned formulas, based on the data available in our dataset.

## Results

### Baseline characteristics

Demographic and psychometric variables are provided in Table 1. Based on MADRS scores at baseline and after the 7th infusion, the 28 patients were classified as responders (*n* = 6) and non-responders (*n* = 22).

**Table 1:** Sociodemographic characteristics of patients^*^ categorized by response to ketamine treatment (defined as at least a 50% reduction in MADRS score). Patients received eight intravenous ketamine infusions at a dosage of 0.5 mg/kg

Variables	Responders (*n* = 6)	Non-responders (*n* = 22)
**Age**		
Mean (SD)	40.2 (11.2)	51.5 (14.0)
**BMI**		
Mean (SD)	26.3 (6.4)	27.6 (4.9)
**Sex**		
Female Male	3 (50%) 3 (50%)	13 (59.1%) 9 (40.9%)
**Education**		
Elementary Vocational Secondary Higher	2 (33.3%) 1 (16.7%) 1 (16.7%) 2 (33.3%)	0 (0%) 2 (9.1%) 9(40.9%) 11 (50%)
**Employment status**		
Unemployed Pensioner Retirement Employed Study	2 (33.3%) 0 (0%) 1 (16.7%) 3 (50%) 0 (0%)	4 (18.2%) 10 (45.5%) 4 (18.2%) 3 (13.6%) 1 (4.5%)
**Marital status**		
Single Informal relationship Married Divorced Widowed	1 (16.7%) 1 (16.7%) 3 (50%) 1 (16.7%) 0 (0%)	5 (22.7%) 1 (4.5%) 11 (50%) 3 (13.6%) 2 (9.1%)
**Concomitant meds**		
TCA SSRI SNRI Other Antipsychotics Mood stabilizers	1 (16.7%) 2 (33.3%) 2 (33.3%) 3 (50%) 0 (0%) 2 (33.3%)	3 (13.6%) 14 (63.6%) 3 (13.6%) 9 (40.9%) 7 (31.8%) 8 (36.4%)
**IDS-SR 30**		
Mean (SD) Range Median 95%CI	46.7 (9.5) 35.0–58.0 47.5 (36.7;56.7)	47.7 (12.5) 27–74 49.0 (42.2;53.3)

BMI – body mass index; IDS-SR 30 - Inventory of Depressive Symptoms-Self Report, 30 item; TCA - tricyclic antidepressants; SSRI - selective serotonin reuptake inhibitor; SNRI - serotonin norepinephrine reuptake inhibitor

^*^ Major depressive disorder patients treated in the Department of Psychiatry of the Medical University of Gdańsk (Poland)

### Dose calculations

Dosage adjustments based on specific formulas for both non-responders and responders are detailed in Tables 2 and 3. Using the Mosteller formula, we first calculated the BSA and then divided the actual administered dose by the BSA. For this calculation, the minimum dose per square meter was 16.77 mg/m² for non-responders and 18.38 mg/m² for responders, with maximum doses of 24.56 mg/m² and 22.62 mg/m², respectively.

**Table 2:** Estimated intravenous ketamine doses in the non-responder group^*^ (*n* = 22), defined as achieving less than a 50% reduction in Montgomery-Åsberg Depression Rating Scale score. Doses were calculated using various formulas, with the percentages of the actual administered dosage relative to body weight indicated in brackets

Patient number	Height (cm)	Weight (kg)	Dose based on actual body mass (mg)^1^	Mosteller^2^	Boer^3^	Devine^4^
Pat1	165	111	55.5	2.26 m^2^ (24.56 mg/m^2^)	28.9 mg (52.0%)	28.5 mg (51.4%)
Pat2	160	83	41.5	1.92 m^2^ (21.61 mg/m^2^)	24.2 mg (58.3%)	26.2 mg (63.1%)
Pat3	164	56	28.0	1.60 m2 (17.5 mg/m2)	21.7 mg (77.5%)	28 mg (100%)
Pat4	179	81	40.5	2.01 m^2^ (20.15 mg/m^2^)	30.8 mg (76.0%)	37.1 mg (91.6%)
Pat5	176	90	45.0	2.10 m^2^ (21.43 mg/m^2^)	32.2 mg (71.6%)	35.7 mg (79.3%)
Pat6	174	100	50.0	2.20 m^2^ (22.73 mg/m^2^)	34.0 mg (68.0%)	34.8 mg (69.6%)
Pat7	176	89	44.5	2.09 m^2^ (21.29 mg/m^2^)	32.0 mg (71.9%)	35.7 mg (80.2%)
Pat8	177	80	40.0	1.98 m^2^ (20.20 mg/m^2^)	27.7 mg (69.5%)	33.9 mg (84.8%)
Pat9	172	85	42.5	2.02 m^2^ (21.04 mg/m^2^)	30.7 mg (72.2%)	33.9 mg (79.8%)
Pat10	162	75	37.5	1.84 m^2^ (20.38 mg/m^2^)	23.6 mg (62.9%)	27.1 mg (72.3%)
Pat11	170	53	26.5	1.58 m^2^ (16.77 mg/m^2^)	22.7 mg (85.7%)	30.7 mg (115.8%)
Pat12	172	90	45.0	2.07 m^2^ (21.74 mg/m^2^)	31.7 mg (70.4%)	33.9 mg (75.3%)
Pat13	188	105	52.5	2.34 m^2^ (22.44 mg/m^2^)	38.9 mg (74.1%)	41.1 mg (78.3%)
Pat14	176	73	36.5	1.89 m^2^ (19.31 mg/m^2^)	28.8 mg (78.9%)	35.7 mg (97.8%)
Pat15	164	90	45.0	2.03 m^2^ (22.17 mg/m^2^)	30.6 mg (68.0%)	30.3 mg (67.3%)
Pat16	165	67	33.5	1.75 m^2^ (19.15 mg/m^2^)	23.3 mg (69.6%)	28.5 mg (85.1%)
Pat17	168	82	41.0	1.96 m^2^ (20.92 mg/m^2^)	25.9 mg (63.2%)	29.8 mg (72.7%)
Pat18	168	72	36.0	1.83 m^2^ (19.67 mg/m^2^)	24.7 mg (68.6%)	29.8 mg (82.8%)
Pat19	169	89	44.5	2.04 m^2^ (21.81 mg/m^2^)	27.0 mg (60.7%)	30.3 mg (91.8%)
Pat20	158	66	33.0	1.70 m^2^ (19.41 mg/m^2^)	21.5 mg (65.2%)	25.3 mg (76.7%)
Pat21	156	57	28.5	1.57 m^2^ (18.15 mg/m^2^)	19.9 mg (69.8%)	24.4 mg (85.6%)
Pat22	164	56	28.0	1.60 m^2^ (17.5 mg/m^2^)	21.7 mg (77.5%)	28.0 mg (100%)

^*^Major depressive disorder patients treated in the Department of Psychiatry of the Medical University of Gdańsk (Poland)

^1^In this method, the dosage was calculated using the 0.5 mg/kg formula

^2^Mosteller formula estimates body surface area (BSA) using the equation: BSA = √[(height (cm) * weight (kg)) / 3600]

^3^Boer formula calculates lean body mass (LBM), representing body weight minus fat weight. LBM dosage calculation differs between males and females. For males, the estimated LBM is 0.407(weight) + 0.267(height) – 19.2, while for females, it is 0.252(weight) + 0.473(height) – 48.3

^4^Devine formula is based on ideal body weight (IBW), an estimate of healthy weight based on height. For males, the equation is IBW = 50 kg + 0.9 kg × (height (cm) − 152); for females, it is IBW = 45.5 kg + 0.9 kg × (height (cm) − 152)

**Table 3:** Estimated intravenous ketamine doses in the responder group* (*n* = 6), defined as achieving at least a 50% reduction in Montgomery-Åsberg Depression Rating Scale score. Doses were calculated using various formulas, with percentages of the actual administered dosage relative to body weight shown in brackets

Patient number	Height (cm)	Weight (kg)	Dose based on actual body mass (mg)^1^	Mosteller^2^	Boer^3^	Devine^4^
Pat1	172	65	32.5	1.76m ^2^ (18.47 mg/m^2^)	24.7 mg (61.8%)	31.6 mg (97.2%)
Pat2	182	68	34	1.85 m^2^ (18.38 mg/m^2^)	28.5 mg (83.8%)	38.4 mg (113.0%)
Pat3	175	100	50	2.21 m^2^ (22.62 mg/m^2^)	34.1 mg (68.2%)	35.3 mg (70.6%)
Pat4	173	67	33.5	1.79 m^2^ (18.72 mg/m^2^)	27.1 mg (81.0%)	34.4 mg (102.7%)
Pat5	160	90	45	2.00 m^2^ (22.50 mg/m^2^)	25.0 mg (74.6%)	26.2 mg (58.2%)
Pat6	158	68	34	1.73 m^2^ (19.65 mg/m^2^)	21.8 mg (64.1%)	25.3 mg (74.4%)

^*^Major depressive disorder patients treated in the Department of Psychiatry of the Medical University of Gdańsk (Poland)

^1^In this method, the dosage was calculated using the 0.5 mg/kg formula

^2^Mosteller formula estimates body surface area (BSA) using the equation: BSA = √[(height (cm) * weight (kg)) / 3600]

^3^Boer formula calculates lean body mass (LBM), representing body weight minus fat weight. LBM dosage calculation differs between males and females. For males, the estimated LBM is 0.407(weight) + 0.267(height) – 19.2, while for females, it is 0.252(weight) + 0.473(height) – 48.3

^4^Devine formula is based on ideal body weight (IBW), an estimate of healthy weight based on height. For males, the equation is IBW = 50 kg + 0.9 kg × (height (cm) − 152); for females, it is IBW = 45.5 kg + 0.9 kg × (height (cm) − 152)

For the Boer and Devine formulas, LBM and IBW were initially computed, followed by dose calculations using a dosage of 0.5 mg/kg. According to the LBM formula, non-responders received between 52.0 and 85.7% of the actual administered dose, and responders received between 61.8 and 83.8%. Using the IBW formula, the estimated doses ranged from 51.4 to 100.0% for non-responders and from 58.2 to 113.0% for responders.

As illustrated in Table 4, nearly all calculations resulted in ketamine underdosing compared to the actual administered dose of 0.5 mg/kg, with only two participants receiving higher doses (102.7% and 113.0%) when using the Devine formula. This discrepancy may be attributed to the fact that a significant portion of our cohort was overweight. Notably, the Devine formula for calculating IBW proved to be the least accurate, with dosing ranges from 58.2 to 113.0% in responders and 51.4–100% in non-responders.

**Table 4:** Minimum and maximum ketamine dosing ranges estimations for responders (*n* = 6) and non-responders (*n* = 22), using alternative formulations to body weight in patients who received eight intravenous ketamine infusions at a dosage of 0.5 mg/kg

Formula	Responders (*n* = 6)	Non-responders (*n* = 22)
Mosteller*	18.38–22.62 mg/m^2^	16.77–24.56 mg/m^2^
Boer**	61.8-83.8%	52.0 − 85.7%
Devine**	58.2-113.0%	51.4 − 100%

*Presented as the actual dose received per square meter of the body surface

**Presented as a percentage of the dose actually received

Participants were individuals diagnosed with major depressive disorder who received treatment at the Psychiatry Department of the Medical University of Gdańsk (Poland)

## Discussion

Regardless of treatment response, the application of alternative ketamine dosing formulas generally resulted in lower doses than the standardized dose of 0.5 mg/kg. This finding indicates a trend toward underdosing with these alternative methods. Notably, only two participants received higher doses when the Devine formula was employed.

Despite the widely recognized consensus guideline on the empirically established ketamine dosage of 0.5 mg/kg [8], researchers are investigating various dosages, including low (0.1–0.2 mg/kg) and high (0.75-1.0 mg/kg) doses, as well as alternative methods such as titration in cases of non-response [12].

Numerous studies support the use of a 0.5 mg/kg dose of IV ketamine. Research indicates that lower doses (0.1–0.2 mg/kg) of ketamine either lack clear or consistent evidence of clinically meaningful efficacy [13, 15] or provide smaller and shorter-lived benefits compared to the 0.5 mg/kg dose [16, 17]. Nonetheless, lower doses (0.2 and 0.4 mg/kg) may be efficacious with the more potent S-isomer, esketamine [18]. While all the aforementioned studies observed a swift resurgence of depressive symptoms after ketamine administration, Fava et al. (2020), in contrast, showcased a more enduring effect at the elevated dosage (1.0 mg/kg), extending up to days 15–30, albeit modest in nature [13]. Furthermore, McIntyre et al. (2020) administered higher doses of 0.75 mg/kg in patients who showed no response to 0.5 mg/kg and demonstrated that response rates improved without a significant increase in adverse effects [2]. Therefore, since the different formulas used in our study predominantly tended to underdose ketamine, they appear to be ineffective.

The dosage range of 0.5-1.0 mg/kg is established through empirical means, although it could stem from calculations based on varying body mass metrics, including adjustments according to BSA. Lipsitz et al. (2022) highlight that most publications do not specify whether dosing relied on ideal or actual body weight. Using IBW for dosing can disadvantage patients with higher BMI, leading to underdosing. The authors emphasize that, due to ketamine’s lipophilic nature, which facilitates crossing the blood-brain barrier, concerns should extend beyond efficacy to include safety considerations [19]. However, an earlier randomized controlled trial revealed no relationship between body weight and the maximum concentration (Cmax) or the area under the curve (AUC) over 6 h for ketamine [20].

Body mass index remains a commonly utilized variable in clinical research but may not adequately capture body composition to fully account for the effects of obesity [21].

Studies investigating the relationship between BMI and ketamine response reveal inconsistencies. A retrospective analysis by Chen et al. (2020) identified obesity as a predictor of favorable response to ketamine treatment [22]. Niciu et al. (2014) reported a positive correlation between BMI and initial response to ketamine, though this did not extend to sustained response [23]. Freeman et al. (2020), despite a small sample size, observed a significantly greater antidepressant response in patients with higher BMI [10]. Conversely, Lipsitz et al. (2022) found no significant differential response to ketamine based on categorical BMI [19]. Dale et al. (2020) demonstrated that BMI did not predict either acute or sustained treatment response and that patients with metabolic syndrome were 50% less likely to respond to acute IV ketamine treatment compared to those without metabolic syndrome [9]. In our study, responders had a lower BMI compared to non-responders, 26.3 versus 27.6, respectively.

A potentially more accurate parameter is the waist-hip ratio (WHR). WHR has been demonstrated to have a strong and consistent association with all-cause and cause-specific mortality [24]. Utilizing WHR could prevent the misclassification of muscular patients, who may have higher body weight, as overweight or obese when using BMI.

Despite certain limitations, the findings of the current study support the practice of calculating the IV ketamine dose based on actual body weight in patients with treatment-resistant MDD. The limitations include a retrospective analysis of data from an open-label, single-center, observational study, a relatively small sample size, the lack of measured ketamine serum concentrations, and the lack of use of continuous variables and multivariate regression analyses. The high proportion of non-responders in this sample exceeds that reported in the available literature, thus limiting the generalizability of the findings. Additionally, the formulas used in this study were derived using only one or two parameters: weight and height. None of the formulas accounted for age or muscle mass. It is well-established that skeletal muscle mass decreases with age; limb muscles in older adults are 25–35% smaller and contain significantly more fat and connective tissue than those in younger individuals [25]. The ages of patients in our study ranged from 24 to 69 years, with a mean age of 40.2 years. Furthermore, the formulas did not consider whether patients engage in regular physical activity, which can result in higher lean body mass (LBM) despite an increase in overall body weight, potentially leading to misclassification of muscle mass as fat tissue. LBM can be more accurately assessed using bioimpedance analysis, which provides personalized measurements, including total body water (TBW) and fat-free mass (FFM) [26]. It may be hypothesized that the adaptive and individualized dosing paradigm may be the one to apply to dissociative rapid-acting antidepressants (RAADs) in general with phasic observation for induction, optimization and maintenance. Thus, the precision-based dosing based on the clinical outcome observed may be far more applicable as compared to the fixed-dose calculations. Interestingly, the analogous dosing strategy may also be observed with study findings for psychedelics in TRD.

Conclusions.

This study suggests that alternative dosing formulations, such as Mosteller, Boer, and Devine, may lead to underdosing when compared to the 0.5 mg/kg IV dose. Additionally, this study supports the adaptive and individualized dosing paradigm for short-term IV ketamine use as an RAAD agent. There is an unmet need for an optimal ketamine dosing formula that ensures both immediate effectiveness and long-term symptom relief. Our study highlights the need for a more individualized dosing approach, especially for patients with a high BMI, to prevent underdosing during ketamine treatment. The unique contributions of this study are particularly valuable for clinicians and provide a foundation for future research aimed at redefining dosing paradigms and incorporating additional parameters in trial designs.

## Data Availability

No datasets were generated or analysed during the current study.
